# Transcriptional Heterogeneity of Oligodendrocytes: Molecular Basis of Diversity Across Development, Brain Regions, and Neurological Diseases

**DOI:** 10.3390/neurolint18060108

**Published:** 2026-06-02

**Authors:** Shingo Miyata, Shoko Shimizu, Yugo Ishino

**Affiliations:** Division of Molecular Brain Science, Research Institute of Traditional Asian Medicine, Kindai University, Sakai, Osaka 590-0197, Japan; shimizu@med.kindai.ac.jp (S.S.); yishino@med.kindai.ac.jp (Y.I.)

**Keywords:** oligodendrocyte, transcriptional heterogeneity, single-cell RNA sequencing, single-nucleus RNA sequencing, disease-associated oligodendrocytes

## Abstract

Oligodendrocytes (OLs) are specialized glial cells essential for the formation and maintenance of the myelin sheath within the central nervous system (CNS). Historically, OLs were considered a functionally homogeneous population. However, the advent and widespread application of single-cell and single-nucleus RNA sequencing (scRNA-seq/snRNA-seq) technologies since 2015 have revealed substantial transcriptional heterogeneity, varying according to developmental stage, anatomical region, and disease state. In this review, we synthesized current advances in the understanding of OL heterogeneity. Nine OL cell classes have been identified in the mouse somatosensory cortex and hippocampal CA1 region, later expanding to 13 distinct subpopulations across ten CNS regions. Furthermore, we characterized disease-associated oligodendrocytes (DAOs)/disease-associated oligodendrocyte lineages (DOLs), identified in various neurological diseases, including multiple sclerosis (MS), Alzheimer’s disease (AD), and spinal cord injury, focusing on their molecular markers, spatial distribution, and pathophysiological roles. We summarized key transcriptional regulatory networks underlying DAO induction, including the signal transducer and activator of transcription (STAT)/interferon regulatory factor (IRF) family, the Yin Yang 1 (YY1)/nuclear factor kappa B (NF-κB) axis, and the SOX9/SOX10 regulatory system. The utility of region-specific brain analyses using spatial transcriptomics (ST) in conjunction with these approaches was also discussed. Finally, we compiled the implications of patient stratification according to white matter glial response patterns derived from large-scale snRNA-seq analyses of patients with progressive MS. Our synthesis shows that oligodendrocytes consist of multiple distinct subtypes that vary across development, brain regions, and disease conditions. In pathological states, they adopt specific disease-associated programs that reflect context-dependent responses and may influence disease progression and repair. This work provides a framework for understanding how oligodendrocyte diversity contributes to neurological disease and may support the development of targeted remyelination therapies.

## 1. Introduction

Oligodendrocytes (OLs) are glial cells restricted to the central nervous system (CNS). By synthesizing the myelin sheath around axons, these cells facilitate the saltatory conduction of action potentials, thereby ensuring rapid and energy-efficient signal transmission within neural circuits [1]. Contemporary research indicates that, beyond traditional myelination, OLs also provide critical metabolic support by supplying substrates such as lactate to axons and actively participate in immune regulation. These findings have fundamentally shifted the perception of OLs from passive structural elements to multifaceted metabolic and immunological regulators [2,3,4,5].

OLs differentiate progressively from oligodendrocyte precursor cells (OPCs), which originate from diverse developmental sources in the subventricular zone during embryogenesis. These precursors are defined by the expression of platelet-derived growth factor receptor alpha (PDGFRα) and neuron-glial antigen 2 (NG2) (also known as chondroitin sulfate proteoglycan 4 [CSPG4]) as surface markers [6,7]. Notably, adult CNS OPCs retain proliferative capacity and serve as the primary reservoir for remyelination following demyelination [8]. The transition from OPCs to mature OLs is a highly orchestrated multi-stage process, encompassing committed OPCs (COPs), newly formed OLs (NFOLs), and myelin-forming OLs (MFOLs), with each stage governed by stage-specific transcription factor networks [9,10,11].

Although the morphological diversity of OLs has been documented since the early 20th century, their functional specialization remained largely overlooked. This paradigm shifted with the emergence of single-cell RNA sequencing (scRNA-seq) and single-nucleus RNA-seq (snRNA-seq) technologies in the field of neuroscience around 2015. By enabling large-scale, single-cell transcriptomic profiling, these techniques uncovered that mature OLs represent a heterogeneous population spanning more than 10 distinct transcriptional states [9,10,11,12,13].

In this review, we aimed to coordinate and summarize these advancements. First, we traced the discovery of OL heterogeneity to its distribution across brain regions and disease states [11]. Second, we evaluated the molecular characteristics and pathophysiological implications of disease-associated oligodendrocytes (DAOs)/disease-associated oligodendrocyte lineages (DOLs) in several neurological disorders [9,14,15]. Third, we examined the transcriptional regulatory mechanisms driving this heterogeneity and summarize current spatial transcriptomics (ST) methodologies [9]. Finally, we discussed clinical insights gained from the molecular stratification of patients with multiple sclerosis (MS) using snRNA-seq and proposed future research directions for remyelination therapies.

## 2. Historical Development of the Discovery of OL Subpopulations

### 2.1. The First Comprehensive Analysis at the Single-Cell Level

The heterogeneity of OLs was first systematically elucidated at the single-cell level in a pivotal study profiling the mouse somatosensory cortex (S1) and hippocampal CA1 region [12]. This landmark scRNA-seq investigation categorized the principal CNS cell types into nine broad classes, further partitioning OLs into six transcriptionally distinct subpopulations: post-mitotic, immature, pre-myelinating, myelinating, intermediate, and terminally differentiated myelinating OLs. Although geographically restricted to two regions, this study was significant, as it was the first to demonstrate that OLs within a single anatomical region show transcriptional diversity corresponding to their maturation stages.

### 2.2. Identification of Comprehensive OL Lineage Subpopulations

The adoption of scRNA-seq has significantly catalyzed a paradigm shift in our understanding of OL lineage diversity. Initial analyses revealed that the OL lineage diverges into substantial transcriptional clusters comprising OPCs, immature OLs, and mature OLs, underscoring that morphological characteristics insufficiently capture the molecular complexity of these cells [12].

Subsequent full-length transcriptomic analysis mapping across diverse CNS regions, including the corpus callosum (CC), cortex, and spinal cord, stratified the lineage into 13 distinct molecular signatures. Specifically, these taxonomies included OPCs (*Pdgfra^+^*, *Ptprz1^+^*), committed oligodendrocyte progenitors (COP; *Gpr17^+^*), early myelinating cells (NFOL/MFOL; *Opalin^+^*, *Mal^+^*), and six mature OL subpopulations (MOL1–MOL6) [9]. Notably, *Il33*, which is highly expressed in MOL5/6, has been suggested to be involved in signal crosstalk between astrocytes and microglia [9,16]. Quantitative analysis of subpopulation distribution across CNS regions in these studies clarified that MFOL2/MOL2 enrichment is characteristic of the CC, whereas MOL5/6 predominate in the spinal cord, demonstrating that OL diversity should be understood as region-specific diversity [17]. Furthermore, transcriptomic analysis of oligodendrocyte lineage cells in the mouse CC has identified distinct gene expression profiles between the juvenile (P14) and adult (P84) stages, further confirming the stage-dependent heterogeneity of OLs [18].

Translational studies have demonstrated that human white and gray matter OLs exhibit differentiation trajectories congruent with those in mice, despite discernible interspecies gene expression divergence [19,20]. Furthermore, 3D brain organoid (hCS) platforms have hinted at human-specific mature OL subtypes [21]. Crucially, it should be noted that the ex vivo transcriptional response accompanying tissue dissociation may influence these results; therefore, subpopulations should be carefully considered when interpreting these results [22]. To mitigate these artifacts, the recent transition to snRNA-seq enabled a high-fidelity reevaluation of OL diversity across the nervous system [23,24].

### 2.3. Application to Human Tissues and Becoming the Standard Method of snRNA-Seq

The advent of snRNA-seq technology has revolutionized the study of human postmortem brain heterogeneity, circumventing the inherent challenges of obtaining live tissue [23,25]. While nuclear RNA may lose some transcriptional information compared to cytoplasmic RNA because of post-transcriptional cytoplasmic transport, stability, and splicing, numerous studies have confirmed a high degree of concordance with scRNA-seq for major cell-type classification. Consequently, snRNA-seq is the standard method for human neurogenomic research.

In MS, snRNA-seq analysis of human white matter lesions has revealed a depletion of mature *RBFOX1^+^*/*OPALIN^+^* OLs, coinciding with the emergence of a pro-inflammatory state characterized by *KLF6/CD44* expression [13]. Parallel studies in experimental autoimmune encephalomyelitis (EAE) mice models identified “immuno-OL” expressing immune-related genes, with similar subpopulations observed in human MS brains [14]. These findings have reframed OLs from vulnerable targets of autoimmunity to active immunomodulators, unveiling potential therapeutic targets. Similarly, in Alzheimer’s disease (AD), snRNA-seq analysis of the prefrontal cortex has dissected early-stage transcriptional shifts in OPCs and confirmed that region-specific transcriptional differences in OL lineage cells are preserved in the human OL lineage [26].

### 2.4. The BRAIN Initiative and Construction of a Whole-Brain Cell Atlas

In the multimodal cell atlas of the mouse primary motor cortex developed by the BRAIN Initiative Cell Census Network, integrative analysis of multiple scRNA-seq/snRNA-seq datasets identified 116 distinct cell types, initially resolving the OL lineage into four OL subtypes (COP, NFOL, MFOL, and MOL) [27]. Subsequent spatial mapping via multiplexed error-robust fluorescence in situ hybridization revealed the presence of a small number of OLs in the gray matter for the first time [27]. Furthermore, the Allen Brain Cell Atlas further subdivided the OL lineage into 99 clusters, demonstrating that the predominant MOL subtype differs based on the type of white matter tracts [28]. Recent breakthroughs in ST have unmasked layer-specific distribution patterns of OL subpopulations in the human dorsolateral prefrontal cortex. Various studies have also documented transcriptional changes in white matter OL subpopulations in schizophrenia and bipolar disorders [29,30,31,32].

**Figure 1:**
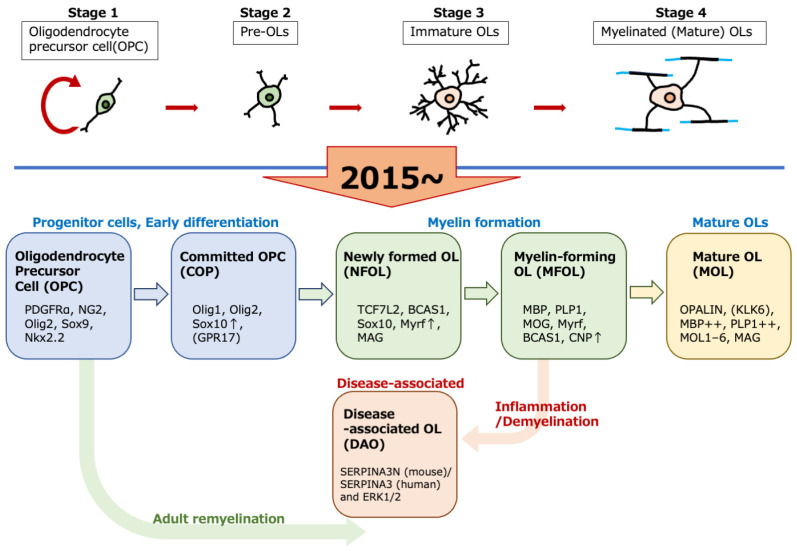
Oligodendrocyte differentiation cascade and stage-specific markers. The figure delineates the multistage differentiation cascade of the OL lineage within the CNS. Before 2015: The upper panel presents that OLs differentiate progressively from OPCs, which originate from various sources within the subventricular zone during embryonic development. This process is recognized as classical morphological OL differentiation. The elliptical arrow in Stage 1 represents the process of self-renewal, whereas the straight arrows from Stage 1 to Stage 4 denote the progression of OL differentiation. After 2015: The lower panel presents the five stages of differentiation, progressing from OPC to committed OPC (COP), newly formed OL (NFOL), myelin-forming OL (MFOL), and culminating in mature OL (MOL). Furthermore, the lower panel displays representative surface markers and stage-specific transcription factors for each differentiation stage. Each arrow represents the progression of OL differentiation. Additionally, the light red arrow indicates pathological pathways in which mature OLs transform into DAOs (Inflammation/Demyelination). The light green arrow indicates the pathways through which OPCs contribute to remyelination following demyelination in the adult CNS (Adult remyelination). This sequential marker transition initiates with PDGFRα/NG2, indicative of OPC identification, and progresses to Olig1/Olig2 at the COP stage, TCF7L2/MAG/BCAS1 at the NFOL stage, Myrf/MBP/PLP1 at the MFOL stage, and OPALIN/*MBP++*/*PLP1++* at the mature MOL stage. SERPINA3N (mouse)/SERPINA3 (human) and ERK1/2 are representative markers of DAO/DOL [7,9,10,32,33]. Abbreviations: PDGFRα, platelet-derived growth factor receptor alpha; Olig2, oligodendrocyte transcription factor 2; Sox9/10, SRY-box transcription factor 9/10; NKX2.2, NK2 homeobox 2; TCF7L2, transcription factor 7-like 2; BCAS1, breast carcinoma amplified sequence 1; Myrf, myelin regulatory factor; MAG, myelin-associated glycoprotein; MBP, myelin basic protein; PLP1, proteolipid protein 1; MOG, myelin-oligodendrocyte glycoprotein; CNP, 2′-3′-cyclic nucleotide 3′ phosphohydrolase; OPALIN, oligodendrocyte specific protein; KLK6, kallikrein-related peptidase 6; SERPINA, serine protease inhibitor A; ERK1/2, extracellular signal-regulated kinase 1/2.

Collectively, these results dismantle the traditional view of mature OLs as a monolithic, homogeneous cell population. It is now well-known that OLs constitute a complex and heterogeneous cellular population encompassing a minimum of 10 distinct transcriptional states [10,11]. These studies are summarized in Figure 1.

## 3. Identification of DAOs/DOLs and Their Pathological Relationship

### 3.1. Overview of DAOs/DOLs

In 2022, several studies identified a novel population of cells termed DAOs/DOLs using single-cell and single-nucleus RNA sequencing analyses [15,33]. While rare in the healthy CNS, these cells expand significantly under pathological stressors, including neuroinflammation, neurodegeneration, and demyelination [15,33].

In a study using AD model mice (5xFAD), DOLs were found to accumulate in proximity to amyloid β plaques, exhibiting a transcriptional profile characterized by the upregulation of 26 genes, including *Serpina3n*, *C4b*, *MHC class I molecules*, *Il33*, and *Klk6* [33]. This transcriptional signature has also been detected in MS, tauopathies, and aging models, indicating that DAOs/DOLs may represent a common cellular response program that is widely conserved across the CNS [15,33].

### 3.2. Three Diseases, AD, MS, and Spinal Cord Injury (SCI)-Associated Subtypes: DA1, DA2, and IFN States

An integrated analysis of mouse models of AD and MS identified three disease (AD, MS, and SCI)-associated activation states within the oligodendrocyte lineage [15]. The first subtype, DA1, represents an inflammation-associated phenotype characterized by upregulated expression of immune signaling genes, including *C4b*, *B2m*, and *Serpina3n*. In contrast, DA2 is associated with activation of the TP53 signaling pathway and cell cycle arrest, rendering these cells more susceptible to apoptosis. The third subtype, termed the IFN state, exhibits increased expression of interferon-stimulated genes (ISGs), such as *Ifit1*, *Ifit3*, and *Mx1* [15,33].

Spatial transcriptomic analysis using a cuprizone-induced demyelination model demonstrated that DA1 cells preferentially accumulated at lesion sites during the remyelination phase. Moreover, these activated OLs spatially co-localize with disease-associated microglia (DAM) and disease-associated astrocytes (DAAs), supporting the existence of a coordinated glial response network under pathological conditions [33].

### 3.3. DAOs in AD—ERK1/2 Signaling and Axonal Myelination Impairment

In an AD mouse model (AppNL-G-F and 5xFAD), a distinct subpopulation of DAOs co-expressing CD74 and myelin basic protein (MBP) was identified. This subpopulation is detectable in the dentate gyrus of the hippocampus as early as three months of age and expands progressively with increasing amyloid-β burden [34]. Functional studies have demonstrated aberrant activation of ERK1/2 signaling in DAOs; notably, pharmacological inhibition of this pathway restored axonal myelination, reduced Aβ deposition, and improved cognitive performance. These findings indicate that DAOs are not merely passively involved but actively contribute to AD pathogenesis [34].

Furthermore, CD74, which is highly expressed in DAOs, has been implicated in the regulation of β-secretase (BACE), suggesting a bidirectional pathogenic interaction between DAOs and amyloid processing pathways [35]. snRNA-seq analysis of human AD brains has also confirmed an increase in the corresponding OL subpopulation and a decrease in the expression of myelin-related genes, thereby supporting the translational relevance of murine observations to human disease [26,36].

### 3.4. DAOs (Oligo_G) in MS

Several snRNA-seq studies of MS brain tissue have identified a distinct oligodendrocyte cluster termed Oligo_G, which lies outside the canonical oligodendrocyte differentiation trajectory [37]. This population was increased in both white and gray matter and was consistently detected across all MS lesion types, including active and chronically inactive lesions, suggesting that MS pathology may extend beyond localized lesions [33,38]. Notably, the ability of mature OLs to transition into a dedifferentiation-like state in response to inflammatory or demyelinating stimuli may be valuable for developing therapeutic strategies to enhance remyelination in MS and other demyelinating diseases.

### 3.5. Cross-Disease Significance and Future Directions of DAOs/DOLs

DAOs/DOLs have been proposed as a novel conceptual framework in glial biology, representing pathology-responsive OL states observed across multiple neurological conditions, including neurodegeneration, demyelination, and neuroinflammation [39,40]. Their core transcriptional signature is conserved across diseases, characterized by the upregulation of complement system genes, MHC class I, and stress response genes, suggesting the activation of a shared oligodendrocyte response program within the CNS under pathological stress [15,33]. However, functional evidence indicates that DAOs/DOLs exhibit context-dependent roles. For instance, while inhibition of ERK1/2 signaling indicates neurotoxic contribution, cytokines such as interleukin-33 (IL-33) have been shown to exert neuroprotective effects. Therefore, DAOs/DOLs cannot be simplistically categorized as either beneficial or detrimental; rather, they likely represent a heterogeneous and dynamic spectrum of functional states, underscoring the need for further mechanistic and temporal studies [41,42].

## 4. Transcription Factor Networks Involved in DAO Regulation

### 4.1. Signal Transducer and Activator of Transcription (STAT)/Interferon Regulatory Factor (IRF) Family

#### 4.1.1. Basic Functions of the STAT Family

The STAT family consists of transcription factors activated by extracellular signals, such as cytokines and interferons, via phosphorylation by Janus kinase (JAK). Upon activation, these factors dimerize and translocate to the nucleus to modulate target gene transcription [43].

Within this axis, STAT1 acts as a central transducer of interferon-gamma (IFN-γ) signaling, exerting a dual role in OL development. Depending on the cellular conditions, OL can either augment or dampen neuroinflammation [44,45]. In DA IFN-OLs, a significant upregulation of ISGs, including *Ifit1*, *Ifit3*, and *Mx1*, was driven by the synergistic activation of STAT1 and STAT2 [15,46,47,48].

#### 4.1.2. Role of the IRF Family

Within the IRF family, IRF3 and IRF7 are key determinants of type I interferon (IFN-α/β) production, governing the innate immune response in OLs [49,50]. Toll-like receptor signaling activates nuclear factor kappa B (NF-κB) and IRF via the adaptor protein MyD88, triggering the induction of cytokines and type I interferons [51,52].

The upregulation of *C4b*, *SERPINA3N*, and *MHC-I* in DAOs supports the activation of an IRF-driven immune transcriptional program [15]. Furthermore, MS snRNA-seq analysis has identified disease-specific transcriptional alterations such as *ASPHD1*, *DBF4B*, *STAT4*, and *ARHGAP27* across OLs and neurons [38].

### 4.2. Yin Yang 1 (YY1) and Histone Deacetylase 1 (HDAC) Complex

#### 4.2.1. The Fundamental Role of YY1

YY1 is a zinc-finger transcription factor in the GLI-Krüppel family that serves as a key regulator of oligodendrocyte lineage differentiation [53]. During the OPC stage, YY1 forms a transcriptional repressor complex with HDAC1, thereby suppressing differentiation-inhibitory genes, including *ID4*, *SOX11*, and *TCF7L2*, which must be downregulated to enable the proper initiation of differentiation [54,55,56]. In mice with oligodendrocyte lineage-specific deletion of Yy1, OPCs remained in an immature state and showed hypomyelination and abnormal derepression of differentiation inhibitors [57].

#### 4.2.2. Interaction with Bone Morphogenetic Protein (BMP) Signaling and Relevance to Pathology

BMP signaling induces the expression of *ID2* and *ID4*, which inhibit OL differentiation by forming inhibitory heterodimers with lineage-specifying transcription factors such as OLIG1/2 and ASCL1 [58,59]. The YY1–HDAC complex counteracts this inhibitory pathway by repressing ID4 expression, thereby facilitating OL differentiation [60]. Although YY1 was initially reported to directly bind to the proteolipid protein (PLP) promoter to activate its transcription as an active component of the complex (acting as an extracellular signal), subsequent evidence has suggested that YY1 may negatively regulate mouse Plp1 [61,62]. Moreover, emerging evidence from an astrocyte study revealed a novel mechanism of YY1 transcriptional regulation involving interactions between YY1 and NF-κB signaling under inflammatory conditions, raising the possibility that YY1 contributes to the regulation of DAO-associated transcriptional programs in pathological states [63,64].

### 4.3. The Roles of the NF-κB Signaling Pathway in OLs

#### 4.3.1. Overview of the NF-κB Pathway

NF-κB functions as a central regulator of inflammatory and cellular stress responses. In OLs, its effects are highly context-dependent, encompassing both cytoprotective and deleterious outcomes [65]. In the canonical pathway, the RelA/p50 dimer modulates the acute inflammatory response, whereas in the non-canonical pathway, NF-κB-inducing kinase (NIK)-dependent processing of p100 to p52 promotes nuclear translocation of the RelB/p52 complex, resulting in sustained transcriptional responses [66].

#### 4.3.2. Protective Roles and the Adverse Effects of Chronic NF-κB Activation

In vitro studies have demonstrated that while NF-κB activation inhibits OL apoptosis, it simultaneously potentiates cytotoxicity induced by tumor necrosis factor alpha (TNF-α), IFN-γ, and reactive oxygen species [67,68]. In EAE models, OL-specific inhibition of NF-κB signaling exacerbates cell death and demyelination, suggesting that NF-κB activation during acute inflammation may serve as a survival signal for OLs [69].

Conversely, chronic activation of NF-κB in mature OLs is thought to exacerbate cell damage. For example, transgenic mice expressing constitutively active IKK2 exhibited gene expression alterations consistent with cellular senescence and white matter degeneration [70,71].

Therefore, the molecular mechanisms underlying these opposing NF-κB responses may be correlated with the senescence-like transcriptional induction observed in specific DAOs [72].

### 4.4. SOX9/SOX10 Transcription Factor Family

#### 4.4.1. The Role of SOX9 and the Transition to SOX10

The SOX-E group transcription factor family (SOX8, SOX9, and SOX10) plays a hierarchical and stage-specific regulatory role in OL lineage development. SOX9 is essential for the initial specification of the OL lineage from neuroepithelial cell progenitors, as demonstrated by the significant reduction in the number of spinal cord OPCs in *SOX9* knockout mice [73,74]. As differentiation progresses, regulatory dominance shifts from SOX9 to SOX10. This transition is mediated by post-transcriptional repression of *SOX9* mRNA by *miR-335* and *miR-338*, which are induced by SOX10 [75]. This control mechanism functions as a near-irreversible molecular switch that stabilizes terminal differentiation [76].

#### 4.4.2. Regulation of Myelin Genes and Associations with Pathology

**Figure 2:**
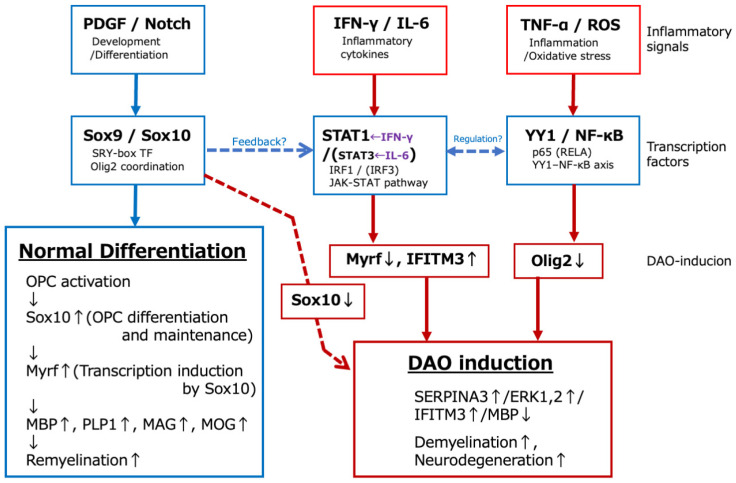
Transcriptional regulatory network generating OL heterogeneity. The transcriptional regulatory network responsible for determining OL heterogeneity under conditions of CNS inflammation and demyelination is depicted as a four-tier hierarchical flowchart. Layer 1 (Upstream Signals): This layer illustrates three types of input—developmental/differentiation signals (PDGF/Notch), pro-inflammatory cytokines (IFN-γ → STAT1/IL-6 → STAT3), and sources of oxidative stress (TNF-α/ROS). Layer 2 (Transcriptional Factor Hubs): Three principal axes of transcriptional control—Sox9/Sox10 (SRY-box transcription factor group), STAT1/STAT3 and IRF1 (IRF3; week) (JAK-STAT pathway), and YY1/NF-κB (p65/RELA axis)—are represented as hub nodes. Layer 3 (Intermediate Effectors): As molecular effects induced by each hub, *Myrf*↓ and *IFITM3*↑ (STAT/IRF axis), *Olig2*↓ (YY1/NF-κB axis), and *Sox10*↓ (Sox9/Sox10 axis) are indicated (DAO induction: differentiation-related genes). Layer 4 (Cellular Phenotype): Two states are depicted: normal continuation of differentiation (*Sox10*↑/*Myrf*↑/*MBP*↑/*PLP1*↑) and DAO induction (*SERPINA3*↑/*ERK1,2*↑/*IFITM3*↑/*MBP*↓) [5,14,15,33,34]. Blue arrows represent the standard differentiation pathway, and red arrows denote the DAO induction pathway initiated by inflammatory signals.

SOX10 cooperates with myelin regulatory factor, forming a core transcriptional complex with OLIG1/2 that is essential for regulating the production of myelin proteins, including *MBP*, *PLP*, myelin-associated glycoprotein (*MAG*), and myelin oligodendrocyte glycoprotein (*MOG*) [77,78]. In contrast, *SOX9* is implicated in reactive astrogliosis and is hypothesized to lie at a lineage bifurcation point between OL and astrocyte fates [79]. Elucidating the broader transcriptional network governing DAOs, including potential alterations in *SOX10* expression, represents a critical research priority, as it is directly linked to therapeutic target identification [75,80]. These studies are summarized in Figure 2.

## 5. Specificity of the Brain Area and Heterogeneity of OLs

### 5.1. MOL2 Is Predominant in the Spinal Cord and Plays a Key Functional Role

scRNA-seq studies have identified at least 12 OL lineage clusters, revealing regional heterogeneity in the distribution of MOL subpopulations across 10 CNS regions [9]. These findings demonstrate that the OL subpopulations are not uniformly distributed, indicating region-specific distribution patterns. Integrated analyses combining ST and smFISH have demonstrated that MOL2 (*Klk6*^+^, *Hopx*+) is significantly enriched in the spinal cord compared to the cerebral cortex, whereas MOL5/6 (*Ptgds*^+^, *Il33*^+^) represents an aging-associated subtype that progressively increases with age across all analyzed regions [17,81]. Within the spinal cord, MOL2 and MOL5/6 exhibit distinct spatial localization patterns, with MOL2 preferentially associated with motor tracts (ventral and lateral funiculi) and MOL5/6 enriched in sensory tracts (dorsal funiculus). These findings suggest a correlation between axonal function and OL subtype specification [17].

In SCI models, MOL2 populations exhibit a more pronounced decrease than that of MOL5/6 in the chronic phase, suggesting that selective loss of this subtype may contribute to impaired remyelination and limited functional recovery [82]. Importantly, OPCs are not intrinsically pre-programmed to differentiate into specific MOL subpopulations; instead, subtype specification is influenced by interactions with local axon-derived signals, extracellular matrix components, and paracrine signals from astrocytes and microglia [17]. These findings are clinically significant, highlighting the potential of targeting endogenous OPC differentiation and modulating the local microenvironment as strategies for promoting regeneration in CNS injury and disease.

### 5.2. Layer-Specific Organization of OLs in the Cortex

Spatial transcriptomic mapping using in situ sequencing (ISS) has revealed a laminar gradient of OLs across the cortex, with mature OLs preferentially accumulating in the deeper cortical layers (layers V–VI) [81]. This distribution reflects the high demand for dense myelination of long-range projection axons in deep cortical regions, including the corticospinal and corticothalamic tracts [83]. Furthermore, in juvenile mice (postnatal day 20 [P20]), a gradient of OL lineage maturation has been observed, progressing from superficial to deeper cortical layers [81]. Early OL maturation in sensory cortical regions may coincide with the establishment of functional neural sensory circuits, consistent with activity-dependent OPC differentiation mechanisms [84,85].

In the CC, myelination persists into adulthood, and accumulating evidence indicates that myelin formation is promoted by motor learning [84,86,87]. Moreover, a subset of cortical neurons demonstrates a discontinuous myelination pattern, in which only selected axonal segments are myelinated, potentially reflecting the functional heterogeneity of OLs within gray matter [88]. In the spinal cord, OL lineage maturation is initiated earlier than that in the brain and follows a caudal-to-rostral temporal gradient [8]. This pattern is thought to parallel the ventral-to-dorsal gradient of neuronal generation observed during spinal cord development [89].

### 5.3. Functional Specialization of OL in the Hippocampus and Visual Cortex

Despite the relatively limited white matter volume in the hippocampus, OLs have emerged as important regulators of memory and cognitive function [90]. Hippocampal OLs exhibit a transcriptional profile distinct from that of somatosensory cortex OLs, notably characterized by expression of *Il33*, a marker associated with MOL5/6, particularly in the CA1 region [12,86,91]. OL-derived IL-33 contributes to synaptic refinement and modulation of the neuroinflammatory response via activation of the ST2 receptor on astrocytes and microglia [92,93]. Furthermore, OLs undergo activity-dependent modifications of myelin during hippocampus-dependent spatial memory tasks, and the survival of OPCs and OLs is essential for the execution of hippocampus-dependent spatial memory tasks [86,91,94].

In the visual cortex, visual experience drives OPC proliferation and differentiation, with activity-dependent myelination contributing to circuit refinement [95,96]. Delayed myelination following dark rearing and accelerated myelin formation with enhanced visual stimulation indicate that visual experience modulates OL subtype specification in the visual cortex [97]. Clinically, the severity of visual impairment in neuromyelitis optica spectrum disorder (NMOSD) and MS may reflect the selective vulnerability of OL subtypes within the visual cortex and optic nerve [98,99,100]. Collectively, these findings, together with layer-specific demyelination patterns observed in cortical lesions, underscore the importance of systematically investigating the relationship between each OL subtype and its functions in the future.

## 6. Integration with Spatial Transcriptomics

### 6.1. Spatial Analysis of MS Lesions

While scRNA-seq and snRNA-seq provide high-resolution transcriptional profiles, they inherently lack spatial information owing to tissue dissociation. Spatial transcriptomics overcomes this limitation by preserving spatial information while quantifying gene expression within intact tissue sections. Its application in OL research has gained increasing attention since the early 2020s. Studies of mouse brain tissue and postmortem human middle temporal gyrus have demonstrated spatial enrichment of DAOs in proximity to amyloid-β plaques [33,101]. Furthermore, spatial co-localization with macrophage/microglia markers (*C1QC*, *C1QA*, and *AIF1*) in AD samples has been identified using ISH techniques [102].

### 6.2. Overview of Spatial Transcriptomics

Integrated analyses combining Visium and snRNA-seq in MS lesions, including chronic active (MS-CA) and chronic inactive (MS-CI) lesions, as well as control white matter, have demonstrated that cell–cell interactions were predominantly restricted to the rim region of MS-CA lesions [103,104]. These interactions involve iron-laden activated microglia, distinct astrocyte subpopulations, and endothelial cells [103,104]. Within the OL lineage, a distinct spatial distribution pattern was evident: mature OLs were significantly reduced in the lesion core, whereas immature OLs and OPCs were enriched in the lesion rim. This resembles that observed with increased DA1 OLs in the cuprizone model and supports the dynamic role of DAO subpopulations in remyelination processes [15,104].

### 6.3. High-Resolution Spatial Mapping and Data Integration

ISS and smFISH enable visualization of the spatial relationships among OL subpopulations, DAM, DAA, and neurons at single-cell resolution [15,105,106]. In an MS study, RNAscope validation has histologically confirmed the spatial expression of patient subgroup-associated genes (*HSP90AA1*, *NAMPT*, *A2M*, *TGFBR*, etc.) identified by snRNA-seq [38]. The integration of ST and snRNA-seq via spatial deconvolution algorithms (e.g., robust cell type decomposition [RCTD, ver. 1.2.0], SPOTlight [ver. 1.4.1], and cell2location [ver.0.1.5]) enabled estimation of OL subpopulation distributions with near single-cell resolution [107,108,109].

## 7. Large-Scale snRNA-Seq Studies in Progressive MS Patients

### 7.1. Discovery of OL Lineage Diversity and Disease-Associated States

snRNA-seq has significantly advanced our understanding of MS pathology by enabling the high-resolution identification of diverse glial and neuronal subpopulations at single-nucleus resolution, a level of detail not achievable with conventional bulk RNA-seq. In postmortem white matter from patients with progressive MS, multiple OL subpopulations have been identified, with distinct subsets showing differential expansion or depletion [13]. These findings challenge the traditional concept of uniform OL vulnerability and have also been observed in normal-appearing white matter (NAWM), demonstrating that MS may be a diffuse disease extending beyond focal demyelinating lesions at the cellular level [13]. Likewise, DAOs were detected in both EAE model mice and human MS brains, suggesting that the principal contributors to MS pathology may include not only immune cells but also OL lineage cells [14].

### 7.2. Cortical Pathology and Glial Phenotypes in Chronically Active Lesions

snRNA-seq analysis of the cortical gray matter has demonstrated that excitatory cut-like homeobox 2 (CUX2)-positive projection neurons exhibit selective vulnerability to meningeal inflammation. Moreover, microglia have been shown to phagocytose myelin-derived transcripts and translocate them into the nucleus [110].

Magnetic resonance imaging (MRI) paramagnetic rim lesions, also referred to as chronic active lesions, have recently emerged as key features in studies of neurodegeneration in progressive MS. MRI-guided snRNA-seq has identified novel glial phenotypes at lesion edges, termed “microglia inflamed in MS (MIMS)” and “astrocytes inflamed in MS (AIMS)” [102,111]. The transcriptomic profile of MIMS overlaps with that of DAM described in other neurodegenerative diseases, with complement C1q being identified as a key mediator of activation. Notably, pharmacological inhibition of C1q in EAE mouse models attenuates disease activity, suggesting that these glial phenotypes represent significant therapeutic targets [102,112].

### 7.3. Largest Integrated Study and Patient Stratification

The largest-scale snRNA-seq study to date, encompassing relapsing-remitting MS (RRMS), secondary progressive MS (SPMS), and primary progressive MS (PPMS), represents a culmination of these efforts [106]. This study classified the OL lineage into OPCs, COPs, and seven OL subpopulations (Oligo_A–G) and delineated differentiation trajectories toward terminally mature OLs (Oligo_D characterized by *MOG+*, *RBFOX1+*, and *KLK6+* expression) [9,38,113]. Importantly, inter-patient variability, rather than lesion type, emerged as the principal determinant of gene expression differences [38]. Moreover, most differentially expressed genes in glial cells were shared across lesion types, suggesting a systemic rather than lesion-restricted disease response [38]. Furthermore, multivariate analysis identified five white matter factors (WM_F1–F5) and stratified patients with MS into four subgroups. Each subgroup was characterized by stress chaperone response, DNA damage response, inhibitory extracellular matrix response, inhibition of immune/regenerative OL response, and regenerative astrocyte morphological alterations [38].

### 7.4. Integrated Understanding and Prospects for Precision Medicine

The principal findings of the International Multiple Sclerosis Genetics Consortium study can be summarized as follows: First, disease-specific transcriptional states were identified across oligodendrocyte lineage cells, microglia, astrocytes, and neurons, with each cell type contributing to neurodegeneration via distinct mechanisms. Second, transcriptional alterations were also observed in NAWM and normal-appearing gray matter, providing molecular evidence for diffuse MS pathology. Third, inter-patient pathological heterogeneity correlates with the cell-type-specific expression of MS susceptibility genes identified through genome-wide association studies, supporting the genetic involvement of OL lineage cells as active participants in the initiation and progression of MS [114].

Collectively, these findings suggest that a significant factor contributing to the uniformly low efficacy rates in numerous progressive MS clinical studies (such as the MS-SMART trial) may be the lack of stratification based on patients’ molecular heterogeneity [115]. The integration of patient subgroup identification using snRNA-seq as a biomarker into future clinical studies may represent a critical step toward precision medicine in MS.

## 8. Conclusions

### 8.1. Paradigm Shift in Oligodendrocyte Research

Over the past decade, research on OLs has undergone a significant shift. Previously regarded as a homogeneous cell population primarily responsible for myelin production, these cells are now recognized as highly diverse and actively engaged in various roles, including disease pathology [9,10,12,13,116]. They initiate distinct genetic programs contingent on the disease type, anatomical location within the brain or spinal cord, and the developmental stage [15]. This transformation was initiated by a revolutionary scRNA-seq analysis [9,12]. Subsequent studies have established criteria for the systematic classification of OL subpopulations and the identification of disease-specific marker genes [13]. These studies are summarized in Table 1.

### 8.2. PDGFRα and Atypical Clinical Manifestations in Oligodendrocytes

PDGFRα, a widely recognized marker of oligodendrocyte precursor cells (OPCs), has recently gained attention for its clinical relevance [117]. Gene amplification and activating mutations of *PDGFRA* are frequently observed in gliomas, particularly diffuse midline gliomas and diffuse intrinsic pontine gliomas, suggesting that OPCs may serve as the cell of origin for malignant glial tumors [118,119,120]. A subset of diffuse intrinsic pontine gliomas (diffuse midline glioma, H3 K27-altered) with *PDGFRA* amplification or mutation exhibits oligodendroglioma-like characteristics and is associated with a particularly poor prognosis, indicating that PDGFRα signaling may function as an early driver of tumorigenesis in OPC-derived gliomas [121]. Furthermore, mutations in NG2/CSPG4, another OPC marker, have been reported to be associated with familial schizophrenia, suggesting that OPC dysfunction may also contribute to the pathology of psychiatric disorders [122,123]. These findings imply that OPC marker molecules are instrumental in identifying the oligodendrocyte lineage and represent significant targets for understanding a range of clinical pathologies.

### 8.3. Cross-Disease Significance of DAOs/DOLs

The concept of DAOs/DOLs has been increasingly identified in MS, AD, Parkinson’s disease, and SCI [15,33]. These findings suggest the existence of a conserved glial activation program shared across diverse neurological disorders.

Key transcriptional regulatory networks governing the induction, maintenance, and resolution of DAOs include the following: (i) STAT/IRF pathway (mediating interferon responses); (ii) NF-κB (context-dependent dual role in protection and injury); (iii) YY1-HDAC complex (regulation of differentiation stages); and (iv) SOX9/SOX10 (cell fate determination in differentiation) [15,48,57,72,73,74,78,80]. Collectively, these pathways orchestrate dynamic transcriptional programs in oligodendrocytes in response to pathological stimuli [38,48,57,60,65,73,124]. These studies are summarized in Table 2.

### 8.4. A Novel Development Through Spatial Transcriptomics

Although scRNA-seq and snRNA-seq enable the high-resolution detection of gene expression in individual cells, they are inherently limited by the loss of spatial context during tissue dissociation. The advent of ST has addressed this limitation. For the first time, the local pathological roles of OL subpopulations and the spatial organization of networks formed by glial cells have been visualized [33].

### 8.5. Fundamental Insights from Large-Scale MS Studies

The largest snRNA-seq study analyzed over 600,000 cell nuclei derived from the brains and spinal cords of patients with MS [38]. The most significant finding of this study was that the heterogeneity of MS pathology is better explained by inter-patient variability than by lesion types (e.g., active or chronic). Moreover, a molecular classification framework was proposed, stratifying patients into four types (WM_F1–F4) based on white matter molecular profiles with high scores [38]. These findings provide a molecular foundation for precision medicine approaches in progressive MS and highlight the potential for integrating molecular stratification into the design of future longitudinal clinical trials and therapeutic interventions.

### 8.6. Transplantation of Platelets and Megakaryocytes and Their Effects on OL Differentiation

Platelets and their precursor cells, megakaryocytes, are increasingly recognized not only for their established roles in hemostasis and thrombosis but also for their contributions to CNS homeostasis and neuroinflammation. Activated platelets release a variety of factors, including PDGF-AA, serotonin, BDNF, and TGF-β [136,137]. These factors are critical signaling molecules involved in OPC proliferation, differentiation, and survival. OPCs express PDGFRα on their surface, and PDGF signaling is crucial for OPC maintenance and proliferation [138,139,140]. Consequently, alterations in the number or activation state of circulating platelets, such as those occurring after therapeutic transplantation, may alter the concentrations of factors such as *PDGF-AA*, potentially affecting the balance of OPC differentiation. Supporting this hypothesis, animal models in which platelets infiltrate the brain parenchyma demonstrate the modulation of neural stem cells and OL lineage precursor cell behavior [141]. Furthermore, in a mouse model of thrombocytosis, sustained platelet hyperaggregation was associated with reduced generation of new OLs, suggesting that quantitative changes in platelets may directly influence OPC differentiation [142]. Additionally, case reports and animal studies have documented unexpected neuropsychiatric symptoms following hematopoietic cell transplantation, indicating that transplanted cells, including platelets and megakaryocytes, may alter the signaling environment in the host brain [143,144]. Collectively, these findings suggest that therapeutic transplantation of platelets and megakaryocytes may modify OPC differentiation via the PDGF pathway and inflammatory cytokines, indicating the importance of considering changes in OL lineage cells when assessing neurological effects post-transplantation.

### 8.7. OL Abnormalities and Psychiatric Disorders, and Therapeutic Implications

Abnormalities in OLs and OPCs have been extensively documented in psychiatric disorders, such as schizophrenia, bipolar disorder, and major depressive disorder, as evidenced by the findings from histological and transcriptomic analyses [29,145,146,147]. Recent psychiatric research has evolved from identifying psychiatric disorders as neuron-exclusive pathologies to recognizing them as network dysfunctions involving diverse cell types, with particular emphasis on OPCs [148,149]. In addition to their traditional role in myelination, OPCs have been implicated in synaptic regulation and neural circuit plasticity, with dysfunction potentially leading to widespread impairment at the neural circuit level [149,150]. In this context, therapeutic approaches targeting OL differentiation hold significant promise as novel intervention strategies for the treatment of psychiatric disorders [145,147,151,152]. Insights into the heterogeneity of OLs and the regulatory mechanisms underlying their differentiation provide a foundation for understanding which OPC subtypes are vulnerable to specific diseases [148,153]. Furthermore, as previously indicated, if therapeutic cell transplantation, particularly the transplantation of platelet or megakaryocyte lineage cells, is able to influence OPC differentiation, this novel approach may induce new research fields for evaluating the unintended effects of existing hematopoietic transplantation therapies on the nervous system or, conversely, actively controlling those effects to potentially intervene in psychiatric symptoms [142]. Although direct clinical evidence is currently limited and this field remains in its early stages, the perspective that links the modulation of the brain environment via platelets or megakaryocytes with OL abnormalities could become a significant issue for the future understanding of psychiatric disease pathophysiology and the therapeutic development of psychiatric diseases.

### 8.8. Summary

Single-cell and spatial transcriptome analyses of OLs represent transformative approaches that provide an unprecedented resolution in understanding neurological disease pathology. This paradigm shift (from the assumption that all patients share the same pathology to understanding diseases and designing treatments based on each patient’s unique molecular profile) is likely to play a central role in bridging next-generation neurological disease research with clinical applications.

## Figures and Tables

**Table 1:** Major scRNA-seq/snRNA-seq studies on OL heterogeneity studies.

Year	Functional Impact	Species	Technology	Region	Cells (*n*)	Subclusters	Key Markers	References
2015	First comprehensive classification of CNS cell types by single-cell RNA sequencing.	Mouse	scRNA-seq	Somatosensory cortex, hippocampal CA1	3005	9 major classes	*Mog*, *Plp1*, *Mag*	[12]
2016	Defined the OL differentiation cascade: OPC-COP-NFOL-MFOL-MOL1–6.	Mouse	scRNA-seq	10 CNS regions	5072	13 subclusters	*Pdgfra*, *Tcf7l2*, *Myrf*, *Opalin*, *Klk6*	[9]
2018	Large-scale validation of broad OL lineage distribution and diversity of developmental CNS.	Mouse	scRNA-seq	Whole brain (39 regions)	160,796	265 clusters	*Mog*, *Plp1*, *Hapln2*	[116]
2018	First definition of disease-associated oligodendrocytes (DAOs).	Mouse	scRNA-seq	Spinal cord (EAE model)	~6000	3 subtypes	*Hspa1a*, *Hspa5*	[14]
2019	Identified human-specific OL subclusters.	Human	snRNA-seq	White matter (temporal lobe)	9948	6 subclusters	*OPALIN*, *MBP*, *PLP1*, *MOG*	[13]
2021	Defined MIMS and AIMS glial phenotypes at chronic active lesion rim; identified C1q as key MIMS activator.	Human	snRNA-seq	Chronic active MS lesions	5000/sample	—	*C1q*, *MIMS-foamy/iron subtypes*	[102]
2022	Identified DAOs across MS, AD, and SCI.	Human	snRNA-seq	Frontal white matter	~80,000	8 subclusters	*HSPA1A*, *IFITM3*, *STAT3*, *MBP↓*	[15]
2025	Stratified progressive MS patients by white matter glial response patterns.	Human	snRNA-seq	Progressive MS white matter (large cohort)	~400,000	OL-high/DAO-high groups	*MBP*, *HSPA1A*, *OPALIN*, *IRF7*	[38]
2022	Systematic integration of OL single-cell studies from 2015–2022.	Mouse/Human	Integrated (scRNA-seq + snRNA-seq)	Multiple regions (integrated)	Integrated meta-analysis	13+ integrated	*Olig2*, *Sox10*, *Myrf*, *Nkx2.2*	Review paper [10]

scRNA-seq, single-cell RNA sequencing; snRNA-seq, single-nucleus RNA sequencing; OL, oligodendrocyte; OPC, oligodendrocyte precursor cell; MOL, mature oligodendrocyte; DAO/DOL, disease-associated OL/DA OL lineage cell; MS, multiple sclerosis; EAE, experimental autoimmune encephalomyelitis; CNS, central nervous system; Mog, myelin-oligodendrocyte glycoprotein; Mag, myelin-associated glycoprotein; Plp1, proteolipid protein 1; Pdgfra, platelet-derived growth factor receptor alpha; Tcf7l2, transcription factor 7-like 2; Myrf, myelin regulatory factor; Opalin, oligodendrocytic myelin paranodal and inner loop protein; klk6, kallikrein-related peptidase 6; Hapln2, hyaluronan and proteoglycan link protein2; Hspa1a/5, heat shock protein family A (Hsp70) member 1A/5; C1q, complement component 1q; IFITM3, interferon-induced transmembrane protein 3; STAT3, signal transducer and activator of transcription 3; MBP, myelin basic protein; IRF7, interferon regulatory factor 7; Olig2, oligodendrocyte transcription factor 2; Sox10, SRY-box transcription factor 10; NKX2.2, NK2 homeobox 2.

**Table 2:** OL subpopulations with transcriptional regulators and signaling pathways.

OL State	Stage	Key Transcription Factors	Regulatory Pathways/Signals	Upstream Inducers	Functional Role	References
OPC. Proliferative phase	Progenitor cell	*OLIG2*, *SOX9*, *NKX2.2*, *ID4*	PDGF-AA	PDGF	Differentiation-promoting	
			Notch	FGF		[7,125]
			Wnt	EGF		
COP. Commitment phase	Fate-committed OPC	*OLIG1*, *SOX10*, *YY1*, *ZFP191*	(GPR17 signaling)	T3 (thyroid hormone)	Differentiation-promoting	[7,9]
			PI3K-Akt	IGF-1		
NFOL. Transition phase	Newly formed OL	*TCF7L2*, *SOX10*, *MYRF*, *NKX6.2*	(Wnt/β-catenin↓)	Contact-dependent signals	Differentiation-promoting	[126,127]
			BMP↓	Laminin		
MFOL. Active myelination phase	Myelinating OL	*MYRF*, *SOX10*, *SP1*, *KLF9*	MYRF autoactivation	Axon-derived signals	Differentiation-promoting	[128,129]
			mTOR	Electrical activity		
				IGF-1		
MOL1/2. Cortex-enriched	Mature OL	*MYRF*, *SOX10*, *OPALIN*	MYRF maintenance activity	Neuronal activity	Differentiation-promoting	[9,130,131]
			Cortical microenvironment	Glutamate		
MOL3/4. Hippocampus-enriched	Mature OL	*MYRF*, *SOX10*, *NR4A1*	Hippocampal microenvironment	BDNF	Differentiation-promoting	[9,85]
			Activity-dependent signaling	Norepinephrine		
MOL5/6. White matter & brainstem	Mature OL	*MYRF*, *SOX10*, *OPALIN*, *HAPLN2*	High MYRF activity	Neuregulin-1	Differentiation-promoting	
			Axon caliber signaling	Axon caliber & length		[7,9]
DAO type I. IFN-responsive	Disease-associated OL	*STAT1*, *STAT3*, *IRF1*, *MYRF↓*, *SOX10↓*	JAK–STAT (activated by IFN-γ)	IFN-γ (primary upstream inducer)	DAO-inducing	
			IFN-γ response (activated by IFN-γ, IL-6, TNF-α)	IL-6; TNF-α (co-inducers of IFN-γ response)		[15,132]
			*→ JAK* *–STAT + IFN-* *γ response co-activation drives DAO induction*			
DAO type II. NF-κB active	Disease-associated OL	*IRF3*, *OLIG2↓*, *YY1↓*	IKK–NF-κB (activated by TNF-α, IL-1β)	TNF-α; IL-1β (primary upstream inducers)	DAO-inducing	
			STING pathway (activated by LPS/ROS)	LPS/ROS (oxidative/innate immune triggers)		[15,57]
DAO type III. SOX9-reactivated	Disease-associated OL	*(SOX9)*, *FOXJ3*, *ATF3*, *SOX10↓*, *MYRF↓*	SOX9–SOX10 antagonism (triggered by ER stress)	ER stress (primary upstream inducer)	Bidirectional	
			Integrated stress response—ISR (triggered by hypoxia, oxidative stress)	Hypoxia; oxidative stress (co-inducers)		[7,14]
Stalled OPC. Remyelination failure—distinct from DAO states	Differentiation-arrested OPC	*YY1, HDAC1/2, DICER1, TCF7L2↓*	Wnt overactivation	Hyaluronan accumulation	Bidirectional	
			Sustained Notch signaling	Inhibitory ECM		[57,133,134,135]
				aging		

OL, oligodendrocyte; OPC, oligodendrocyte precursor cell; COP, committed OPC; NFOL, newly formed OL; MFOL, myelin-forming OL; MOL1–6, mature OL subtypes 1–6; DAO, disease-associated oligodendrocyte; Olig2, oligodendrocyte transcription factor 2; Sox9/10, SRY-box transcription factor 9/10; YY1, Yin Yang 1; NKX2.2/6.2, NK2 homeobox 2/6.2; TCF7L2, transcription factor 7-like 2; Myrf, myelin regulatory factor; KLF9, Krüppel-like factor 9; NR4A1, nuclear receptor subfamily 4 group A member 1; HAPLN2, hyaluronan and proteoglycan link protein 2; STAT1/3, signal transducer and activator of transcription 1/3; NF-κB (p65/RELA), nuclear factor kappa B subunit p65; IRF1/3, interferon regulatory factor 1/3; FOXJ3, forkhead box J3; ATF3, activating transcription factor 3; HDAC1/2, histone deacetylase 1/2; DICER1, dicer 1 ribonuclease III; mTOR, mechanistic target of rapamycin; ISR, integrated stress response; ECM, extracellular matrix. For DAO type I: IFN-γ functions as the primary upstream inducer that activates both the JAK–STAT signaling pathway and the IFN-γ response; IL-6 and TNF-α act as co-inducers amplifying the IFN-γ response. Cooperative activation of JAK–STAT and the IFN-γ response collectively drives DAO type I induction. For DAO type II: TNF-α and IL-1β are upstream inducers of IKK–NF-κB signaling, while LPS and ROS activate the STING pathway. For DAO type III: ER stress, hypoxia, and oxidative stress act as upstream stressors triggering SOX9–SOX10 antagonism and ISR. A downward arrow indicates a reduction in the expression of each gene.

## Data Availability

No new data were created or analyzed in this study.
